# Effects of Acute Dietary Polyphenols and Post-Meal Physical Activity on Postprandial Metabolism in Adults with Features of the Metabolic Syndrome

**DOI:** 10.3390/nu12041120

**Published:** 2020-04-17

**Authors:** Dustin W Davis, James W Navalta, Graham R McGinnis, Reimund Serafica, Kenneth Izuora, Arpita Basu

**Affiliations:** 1 Department of Kinesiology and Nutrition Sciences, School of Integrated Health Sciences, University of Nevada, Las Vegas, NV 89154, USA; dustin.davis@unlv.edu (D.W.D.); james.navalta@unlv.edu (J.W.N.); graham.mcginnis@unlv.edu (G.R.M.); 2 School of Nursing, University of Nevada, Las Vegas, NV 89154, USA; reimund.serafica@unlv.edu; 3 Department of Internal Medicine, School of Medicine, University of Nevada, Las Vegas, NV 89154, USA; kenneth.izuora@unlv.edu

**Keywords:** overweight, obesity, dysglycemia, dyslipidemia, oxidative damage, inflammation, exercise, oils, fruits, teas, legumes

## Abstract

Approximately 22% of U.S. adults and 25% of adults globally have metabolic syndrome (MetS). Key features, such as dysglycemia and dyslipidemia, predict type 2 diabetes, cardiovascular disease, premature disability, and death. Acute supplementation of dietary polyphenols and post-meal physical activity hold promise in improving postprandial dysmetabolism. To our knowledge, no published review has described the effects of either intervention on postprandial glucose, insulin, lipids, and markers of oxidative damage and inflammation in adults with features of MetS. Thus, we conducted this review of controlled clinical trials that provided dietary polyphenols from oils, fruits, teas, and legumes during a dietary challenge, or implemented walking, cycling, and stair climbing and descending after a dietary challenge. Clinical trials were identified using ClinicalTrials.gov, PubMed, and Google Scholar and were published between 2000 and 2019. Dietary polyphenols from extra virgin olive oil, grapes, blackcurrants, strawberries, black tea, and black beans improved postprandial glucose, insulin, and markers of oxidative damage and inflammation, but results were not consistent among clinical trials. Freeze-dried strawberry powder distinctly improved postprandial insulin and markers of oxidative damage and inflammation. Post-meal physical activity attenuated postprandial glucose, but effects on postprandial lipids and markers of oxidative damage and inflammation were inconclusive. Consuming dietary polyphenols with a meal and completing physical activity after a meal may mitigate postprandial dysmetabolism in adults with features of MetS.

## 1. Introduction

An estimated 30.3 million adults (9.4%) in the United States (U.S.) are presently diagnosed with type 2 diabetes (T2D) [1], and approximately 22% meet the criteria for metabolic syndrome (MetS) or pre-diabetes [2]. The burgeoning prevalence of T2D is a grave public health concern. A prudent prevention approach is to target the groups at the greatest risk of developing T2D, such as those with MetS, and to identify interventions that address its pathological mechanisms. Different organizations define MetS slightly differently, but all definitions include measures of obesity, dysglycemia, dyslipidemia, and hypertension. A commonly used definition is the one revised in 2005 by the National Cholesterol Education Program Adult Treatment Panel III [3]. According to this definition, classification of MetS requires the presence of at least three of the following conditions: visceral adiposity indicated by an increased waist circumference (>40 and >35 inches (in) in males and females, respectively); dysglycemia as indicated by an elevated fasting plasma glucose (≥100 milligrams (mg)/deciliter (dL), or on medications to lower blood glucose); dyslipidemia as indicated by a low plasma concentration of high-density lipoprotein cholesterol (HDL-C, <40 and <50 mg/dL in males and females, respectively) or elevated fasting plasma triglycerides (TGs ≥ 150 mg/dL, or on medications to lower lipids); and elevated resting blood pressure (>130 millimeters of mercury (mmHg) systolic or >85 mmHg diastolic, or on medications to lower blood pressure) [3,4,5]. Importantly, the MetS criteria are associated with an increased risk of developing T2D, cardiovascular disease (CVD), and all-cause mortality [6]. Successfully addressing early-stage metabolic disorder in individuals with MetS may dramatically attenuate the personal and societal burden of T2D in the coming decades.

### 1.1. Postprandial Dysmetabolism

Ingesting energy-containing foods and beverages challenges the body to digest and absorb carbohydrates, lipids, and proteins and transport them to peripheral tissues (i.e., hepatic, adipose, and skeletal muscle) for usage or storage. This challenge is exacerbated in people with MetS and T2D. While the pathophysiology of postprandial dysmetabolism has been excellently reviewed elsewhere [7], a brief description and summary of its importance are provided. A decline in pancreatic β-cell function, increased peripheral insulin resistance, and a reduced systemic lipoprotein lipase activity promote visceral adiposity as well as exaggerated postprandial glucose (PPG) and postprandial lipid (PPL) excursions [8,9,10,11,12,13]. Prolonged elevations in PPG stimulate the formation of advanced glycation end products and reactive oxygen species that cause oxidative damage and inflammation in the arterial wall [14]. Furthermore, prolonged PPL, which commonly manifests as postprandial hypertriglyceridemia, induces the expression of pro-inflammatory cytokines, cellular adhesion molecules, and leukocytes that contribute to the formation of fatty plaques that narrow arteries [14,15]. These processes are exacerbated by the frequent eating pattern common among U.S. adults. Repeated postprandial challenges mean less time is spent in the post-absorptive state and that PPG and PPL excursions are virtually perpetual. This phenomenon poses a grave risk to public health. Steep and prolonged elevations in PPG are linked to CVD, even at levels below the diabetic range [16,17,18,19,20,21]. Furthermore, abnormal PPG predicts CVD-related mortality better than fasting plasma glucose [22,23]. Abnormal elevations in PPL are linked to CVD and CVD-related mortality, independent of plasma glucose [15,24,25]. Therefore, targeting exaggerated PPG and PPL is vital in managing MetS and mitigating its progression to T2D and CVD.

### 1.2. Preventative Roles of Dietary Polyphenols and Physical Activity

The Diabetes Prevention Program demonstrated the importance of diet, physical activity, and metformin in preventing the progression of impaired fasting glucose (IFG) or impaired glucose tolerance (IGT) to T2D [26]. Importantly, the behavioral changes related to both diet and physical activity were more effective in reducing the incidence of T2D than metformin [26]. This finding illustrates the critical need for individuals at risk of T2D to improve their diet and level of physical activity.

Dietary choices are a key factor that influence IFG, IGT, and health across the lifespan. Dietary polyphenols are one of many dietary compounds that have received attention for their health benefits and potential to improve postprandial metabolism [27,28,29,30]. In vitro and in vivo studies with animals have shown that polyphenolic compounds inhibit carbohydrate digestion and absorption, thereby dampening PPG excursions [27]. Additionally, randomized controlled clinical trials with humans have shown that chronically consuming dietary polyphenols improves insulin sensitivity, PPG, and PPL in overweight or obese adults with at least one feature of MetS [28,31]. In other controlled clinical trials that acutely supplemented dietary challenges with dietary polyphenols from various sources (extra virgin olive oil, teas, apple peels, blackberries, blackcurrant, strawberries, and red wine), dietary polyphenols attenuated satiety, PPG, oxidative stress, and inflammation, but these findings were not consistent and have are largely been gathered in samples of healthy adults [32,33,34,35,36,37]. These clinical trials illuminate a notable gap in the literature: it remains to be elucidated how acute dietary polyphenol supplementation with a dietary meal challenge affects PPG, PPL, and markers of oxidative damage and inflammation in adults with features of MetS.

In addition to dietary modifications, modifying habits surrounding physical activity is vital in at-risk individuals. By its definition, physical activity necessitates the contraction of skeletal muscle, which rapidly induces the translocation of transport proteins, primarily glucose transporter type 4 (GLUT4), to the surface of skeletal muscle cells in both healthy individuals and those who are obese and have T2D [38,39,40,41,42]. Contraction-induced GLUT4 signaling follows pathways largely independent of the insulin-induced pathways, and is thus retained after the development of peripheral insulin resistance [43]. Another benefit of physical activity is that it acutely sensitizes skeletal muscle to insulin for up to 48 hours (h) after physical activity, and chronic exercise training improves overall insulin sensitivity [43]. These transient and chronic adaptations by skeletal muscle provide mechanistic justification for including physical activity in any lifestyle intervention intended to improve glycemic control. Clinical trials have investigated the effects of acute bouts prior to [44,45,46] and after [47,48] a dietary challenge on PPG, and day-prior physical activity on next-day PPL [49,50]. The strongest evidence for the efficacy of physical activity in blunting PPG is a recent review of clinical trials that had adults with T2D complete exercise ≤ 3 hours after a dietary challenge [51]. While these findings are promising, a gap remains: it has not yet been determined which modalities, intensities, and durations of physical activity best improve PPG, PPL, and markers of oxidative damage and inflammation after a dietary challenge in adults with MetS who do not yet meet the diagnostic criteria of T2D.

In summary, several published clinical trials have examined how acute and chronic dietary polyphenol supplementation and acute bouts of physical activity may benefit postprandial metabolism in people who are healthy and those who have T2D. A relatively smaller number of clinical trials and seemingly no reviews have reported the effects of acute dietary polyphenol supplementation or acute post-meal physical activity on postprandial metabolism in adults with features of MetS only. This information would be of value to health professionals in making recommendations for prophylactic lifestyle modification. Therefore, we conducted this comprehensive review of controlled clinical trials with the primary aim of answering two unresolved questions: (1) Which forms of acute dietary polyphenol supplementation and post-meal physical activity efficaciously attenuate PPG and PPL excursions in MetS? and (2) Do any of the identified interventions concomitantly reduce circulating markers of oxidative damage and inflammation associated with MetS? Our secondary aim was to use the findings to recommend changes to the current U.S. dietary and physical activity guidelines for adults.

## 2. Materials and Methods

The literature search focused exclusively on controlled clinical trials investigating the use of acute supplementation of dietary-polyphenol-containing foods, beverages, and supplements or post-meal physical activity in adults with features of the MetS. Outcome measures of interest were postprandial glucose, insulin, lipids, and markers of oxidative damage and inflammation. All searches were conducted in PubMed, Google Scholar, and the U.S. Library of Medicine repository for clinical trials (https://www.clinicaltrials.gov/) and were limited to clinical trials published between 2000 and 2019. Key words included “metabolic syndrome”, “prediabetes”, “postprandial”, “polyphenols”, “berries”, “physical activity”, “exercise”, “exercise therapy”, “plasma glucose”, “lipids”, “triglycerides”, “LDL cholesterol”, “HDL cholesterol”, “lipoproteins”, “oxidative damage”, and “inflammation.” Inclusion criteria were clinical trials with control groups and standardized dietary challenges; clinical trials supplementing dietary polyphenols with a single dietary challenge; clinical trials implementing physical activity during the postprandial period (i.e., post-meal, only immediately or nearly immediately after a dietary challenge); and clinical trials that measured postprandial glucose, insulin, lipids, and markers of oxidative damage and inflammation at baseline and after the dietary challenge linked directly with the dietary or physical activity intervention. Exclusion criteria included not having a control group; evaluating non-human animals; having human participants with CVD, T2D, or renal disease; and investigating the postprandial effects on metabolism of physical activity occurring outside of the postprandial period (i.e., the evening prior to the dietary challenge or before the dietary challenge on the day of the dietary challenge). The first author and corresponding author independently conducted the search and then compiled the articles for data extraction.

## 3. Results

**Figure 1:**
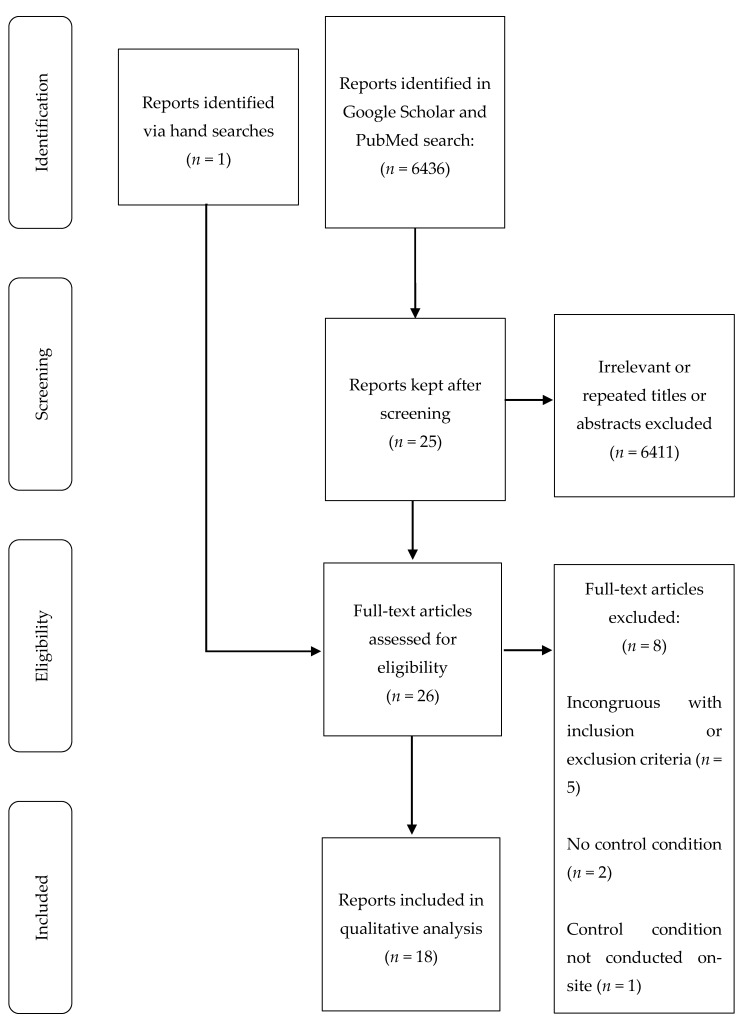
Flow diagram depicting the process of reviewing reports for inclusion.

Our initial searches provided 6436 reports. The authors narrowed the selection using the inclusion and exclusion criteria (Figure 1). The 18 reports included in this review are described below in Tables 1 and 2. Unless otherwise stated, the tabular and in-text data are expressed as means ± standard deviations (SD).

### 3.1. Demographics

The distribution of the features of MetS among participants in the clinical trials was as follows: eleven clinical trials (twelve reports) included participants who met the MetS criterion for abdominal obesity (body mass index (BMI) ≥ 30.0 kilograms [kg]/m^2^, waist circumference > 40 in/102 centimeters (cm) for males and >35 in/88 cm for females) [52,53,54,55,56,57,58,59,60,61,62,63]; twelve clinical trials (thirteen reports) included participants who either met the MetS criterion for fasting dysglycemia or had IGT (blood glucose ≥ 140 mg/dL 2 h after oral glucose tolerance test (OGTT)) [52,55,56,57,58,59,60,61,62,64,65,66,67]; five clinical trials (six reports) included participants who met the MetS criterion for fasting dyslipidemia [58,59,60,63,64]; and five clinical trials (six reports) included participants who met the MetS criterion for elevated blood pressure [53,55,57,58,60,61].

### 3.2. Effects of Dietary Oils, Fruits, Teas, and Legumes on Postprandial Glucose, Insulin, and Lipids

Nine clinical trials evaluated the effects of supplementing a dietary challenge with dietary polyphenols from oils, fruits, teas, and legumes on postprandial metabolism in adults with features of MetS (Table 1). The dietary polyphenols were obtained from extra virgin olive oil [52], grapes [53,58], resveratrol and curcumin powder [54], strawberries [56,68,69], blackcurrant [59], black tea [64], and black beans (one clinical trial with two reports) [55,57]. In seven of the clinical trials, the dietary polyphenols were consumed with a dietary challenge [52,53,54,55,56,57,58,68,69]. The two other clinical trials utilized dietary challenges comprising either sucrose in water [64] or sucrose with cream [59].

Only Carnevale et al. tested the postprandial effects of extra virgin olive oil and reported participants’ apolipoprotein B48 (ApoB48) concentrations to be significantly lower at 2 h with the oil compared to the control phase in obese adults with IFG (−16.7%, *p* < 0.05) [52]. In contrast, numerous clinical trials have investigated the postprandial effects of various fruits. Bardagjy et al. tested 60 grams (g) whole grape powder in obese adults, many of whom had MetS, and did not find significant differences in 5-h glucose, insulin, or triglyceride (TG) incremental area under the curve (iAUC) compared to the control phase (*p* > 0.05) [53]. In another clinical trial, Edirisinghe et al. reported that 300 mg grape seed extract reduced 6-h glucose area under the curve (AUC) by 3.5% (*p* < 0.05) but did not affect 6-h insulin, TG, or total cholesterol (TC) AUC (*p* > 0.05) compared to the control phase [58]. Vors et al. administered two capsules (total of 200 mg resveratrol (found in grapes) and 100 mg curcumin (found in turmeric)) to adults and did not find significant differences in 6-h glucose, insulin, or TG iAUC compared to the control phase (*p* > 0.05) [54].

In addition to grapes, strawberries were another fruit evaluated in several clinical trials. Park et al. did not find report differences in glucose after administering freeze-dried strawberries to obese adults with IFG (*p* > 0.05) [56]. The 40 g dose reduced 6-h insulin by ~12% compared to the 10 g dose and control phase (*p* < 0.05) [56]. Additionally, the 40 g dose blunted the insulin to glucose ratio, insulin absolute peak, and insulin incremental increase from baseline (*p* < 0.05) [56]. None of the doses affected 6-h TG concentrations (*p* > 0.05) [56]. Similarly to Park et al. [56], Edirisinghe et al. provided a 10 g dose of freeze-dried strawberries to overweight adults with hyperlipidemia and did not report an effect on postprandial glucose (*p* > 0.05) [68]. Unlike in the trial conducted by Park et al. [56], the 10 g dose reduced 6-h insulin by 12% compared to the control phase (*p* = 0.01) [68]. In an analysis of the same trial participants reported on by Edirisinghe et al. [68], Burton-Freeman et al. found that the 10 g freeze-dried strawberries reduced 6-h TG by 3.7% compared to the control phase (*p* = 0.006) [69]. Additionally, 6-h low-density lipoprotein cholesterol (LDL-C) was 2.5% higher after freeze-dried strawberries compared to the control phase (*p* < 0.05) [69]. Only one other type of fruit, blackcurrant, was tested in a clinical trial meeting our inclusion criteria. Huebbe et al. provided a 250 g blackcurrant beverage to obese males with IFG and elevated fasting TG and did not observe postprandial differences in glucose, insulin, TG, TC, LDL-C, or HDL-C compared to the control phase (*p* > 0.05) [59].

In addition to dietary polyphenols sourced from oils and whole fruits, tea polyphenols were evaluated by one clinical trial. Butacnum et al. administered 500 milliliters (mL) of black tea, containing differing concentrations of black tea polymerized polyphenols, to adults with pre-diabetes [64]. Compared to the control phase, the 110 and 220 mg black tea polymerized polyphenols reduced the 1-h and 1.5-h glucose iAUC compared to the control phase (12%–15% reduction with 110 mg and 220 mg black tea polymerized polyphenols vs. control phase at 1.5 h, *p* < 0.05) [64]. The glucose response did not significantly differ between the 110 and 220 mg concentrations (*p* > 0.05) [64]. Postprandial insulin did not significantly differ after black tea polymerized polyphenols (*p* > 0.05), and lipid data were not reported [64].

The final source of dietary polyphenols evaluated by clinical trials in this review is black beans. Reverri et al. did not find a significant difference in postprandial glucose when adults with MetS consumed a black bean meal, added-fiber meal, and no-fiber meal on postprandial glucose (*p* > 0.003) [55]. Though glucose did not differ, 5-h insulin was lower after the black bean meal compared to the added-fiber and no-fiber meals (~50% lower than the no-fiber meal, *p* < 0.0001) [55]. A separate report on the same clinical trial stated that postprandial TG did not differ among the meals (*p* > 0.05) [57].

### 3.3. Effects of Dietary Oils, Fruits, Teas, and Legumes on Postprandial Markers of Oxidative Damage and Inflammation

In the single trial that evaluated extra virgin olive oil, Carnevale et al. reported that the oil resulted in lower lipopolysaccharide (LPS), oxidized LDL (OxLDL), and soluble Nox2-derived peptide (sNox2-dp) at 1 and 2 h compared to the control phase [52]. Specifically, at 2 h after oil supplementation, the concentrations of LPS, OxLDL, and sNox2-dp concentrations were approximately 37.5%, 57.1%, and 42.1% lower, respectively, compared to the control phase (*p* < 0.001, *p* < 0.001, and *p* < 0.05, respectively) [52].

Markers of oxidative damage and inflammation were also commonly reported in the clinical trials with grape polyphenols. Bardagjy et al. reported lower endothelin-1 (ET-1) at 5 h (−13.33%) after 60 g grape powder compared to the control phase (*p* < 0.05) [53]. The same clinical trial did not reveal significant differences in interleukin-6 (IL-6), monocyte chemoattractant protein-1 (MCP-1), OxLDL, plasminogen activator inhibitor-1 (PAI-1), retinol-binding protein 4, soluble intercellular adhesion molecule-1 (sICAM-1), soluble vascular cellular adhesion molecule-1 (sVCAM-1), or tumor necrosis factor (TNF) concentrations (*p* > 0.05) [53]. Edirisinghe et al. reported similar null findings after supplementing grape seed extract, which did not affect 6-h IL-6, TNF-α, or lipophilic oxygen radical absorbance capacity (ORAC) iAUC compared to the control phase (*p* > 0.05) [58]. In contrast, the 6-h hydrophilic ORAC iAUC was 196% higher after the grape seed extract compared to the control phase (*p* < 0.05) [58]. OxLDL was reduced by approximately 8.3% at 5 h from baseline after the grape seed extract, but not in the control phase (*p* < 0.01) [58]. In contrast to the 60 g grape powder supplementation by Bardagjy et al. [53], resveratrol and curcumin supplementation by Vors et al. caused a 4643% reduction in 6-h sVCAM-1 iAUC compared to the control phase (*p* = 0.01). The 6-h IL-6, interleukin 8, MCP-1, C-reactive protein (CRP), sICAM-1, or soluble endothelial selectin (sE-selectin) iAUC did not differ (*p* > 0.05) [54].

Several trials also indicated that strawberry polyphenols reduced the postprandial expression of some markers of oxidative damage and inflammation. After a 20 g dose of freeze-dried strawberries, Park et al. reported a reduction in 6-h OxLDL (normalized to fasting) compared to the control phase, a 10 g dose, and 40 g dose (3100% greater after 20 g vs. 0 g, *p* < 0.05) [56]. Effects were not observed for 6-h IL-6 or ORAC [56]. Edirisinghe et al. reported that 10 g freeze-dried strawberries resulted in lower 6-h IL-6 (−16.1%, *p* = 0.05) and 6-h high-sensitivity CRP (hs-CRP, −12.9%) concentrations compared to the control phase (*p* = 0.05) [68]. The 6-h PAI-1, TNF-α, and interleukin-1β (IL-1β) concentrations did not differ after the freeze-dried strawberries compared to the control phase (*p* > 0.05) [68]. Burton-Freeman et al. separately reported that 10 g freeze-dried strawberries resulted in a lower 6-h OxLDL concentration (normalized to fasting, −730.0%) only in male participants compared to their control phase (*p* = 0.0008) [69].

In addition to the clinical trials with oil, grapes, and strawberries, two other clinical trials investigated the effects of blackcurrant and black beans on postprandial changes in markers of oxidative damage and inflammation. After administering a 250 g blackcurrant beverage, Huebbe et al. observed an 85.2% higher IL-6 concentration at 4 h compared to baseline (*p* = 0.009), but no change from baseline was observed with the control phase (*p* = 0.285) [59]. The researchers also observed lower IL-1β and TNF-α concentrations (both ex vivo) at 4 h compared to baseline with the control phase, but these changes were not retained in vivo in the systemic circulation (*p* = 0.09 and *p* = 0.08, respectively). The 2- and 4-h ORAC AUC was greater with blackcurrant compared to the control phase (2 h: 8.6% greater, *p* = 0.006; 4 h: 12.8% greater, *p* = 0.008), as was the 4-h ascorbic acid AUC (4 h: 12.4% greater, *p* = 0.037) [59]. Overall postprandial responses in IL-6, IL-1β (ex vivo), OxLDL, α-tocopherol, and paraoxonase did not significantly differ between the blackcurrant and the control phases (*p* > 0.05) [59]. Reverri et al. also reported null findings after supplementing a black bean meal, fiber-matched meal, and an antioxidant-matched meal. Postprandial IL-6, OxLDL, sICAM-1, or sVCAM-1 (*p* > 0.006) did differ among the treatments [57].

### 3.4. Effects of Physical Activity on Postprandial Glucose, Insulin, and Lipids

Seven clinical trials evaluated the postprandial effects of walking [60,61,62], cycling [63,66], and stair climbing and descending [65,66,67] (Table 2). Six of the seven clinical trials implemented physical activity after a dietary challenge in the form of foods or beverages [60,61,62,63,66,67], and one implemented physical activity after a 75 g dextrose-tolerance test [65]. Two clinical trials initiated physical activity immediately after the participants consumed the meal [60,62], and two other clinical trials initiated physical activity 0.5 h after the dietary challenge [61,65]. One clinical trial initiated physical activity 1 h from the end of the dietary challenge [63], and another two clinical trials initiated physical activity 1.5 h from the start of the dietary challenge [66,67].

Among the types of physical activity implemented, walking required the lowest intensity of exertion. Based on data reported in three clinical trials, acute postprandial walking exersts a favorable effect on postprandial glucose metabolism. DiPietro et al. reported that in older, sedentary, and obese adults with IFG, three 15 min walks (beginning 0.5 h after breakfast, lunch, and dinner, respectively) blunted 24-h AUC by 15.9% (*p* < 0.05) and 3-h post-dinner AUC by 7.7% (*p* < 0.05) compared to an inactive control phase [61]. Insulin was only measured during the control phase in this clinical trial, precluding an evaluation of the insulin response to walking. Lunde et al. reported that, in obese females with IGT, 20 and 40 min of walking immediately after eating reduced 2-h glucose iAUC by 30.6% (*p* = 0.025) and 39.0% (*p* = 0.006), respectively, compared to an inactive control phase [62]. Furthermore, the 20 and 40 min walks dampened peak glucose by 8.2% and 16.3%, respectively, though the reduction was only significant after the 40 min walk (*p* = 0.001) [62]. Insulin data were not reported. Data from a third clinical trial contrast with the aforementioned findings. Diekmann et al. reported a higher concentration of glucose in older, obese adults with IFG when the participants walked for 30 min immediately after eating compared to an inactive control phase (difference observed at 1.5 h, *p* < 0.001) [60]. Despite the difference in blood glucose at that specific time point, the 4.5-h glucose AUC did not differ (*p* > 0.05) [60]. Though the insulin concentration was lower at the 3-h time point after walking compared to an inactive control phase, the 4.5-h insulin AUC did not differ (*p* > 0.05) [60]. In the same clinical trial, data were reported for repeated postprandial TG measurements and 4.5-h non-esterified fatty acid (NEFA) AUC, but differences between walking and the inactive control phase were not significant (*p* > 0.05) [60].

Another form of post-meal physical activity evaluated in this review is cycling on an ergometer. Derave et al. reported data from sedentary adult males with MetS who completed 45 min of cycling at ~60% of their respective maximal relative oxygen consumption (VO_2max_), 1 h after starting breakfast. Blood glucose declined by 35.5% from immediately before physical activity to the end of the bout (*p* < 0.05). At 15 min after physical activity, blood glucose remained 21.0% lower than immediately before physical activity (*p* < 0.05) [63]. While not significant, blood insulin decreased by 81.6% from immediately before physical activity to the end of the bout before rebounding to 43.9% of the before physical activity concentration by 15 min after physical activity (*p* > 0.05) [63]. Reported TG iAUC data did not reveal significant differences between cycling and an inactive control phase (*p* > 0.05) [63]. Takaishi and Hayashi had adults with IGT cycle for ~8 min at ~60%–65% heart rate reserve (HRR), 1.5 h after starting breakfast. With cycling activity, participants’ blood glucose was lower at 1.75 and 2 h compared to when they completed an inactive control phase (1.75 h: −20.2%; 2 h: −19.4%), although these differences were not significant (*p* > 0.05) [66]. Insulin data from this clinical trial were only reported for the collective sample that comprised people with IGT and people with diagnosed T2D. Therefore, the present review cannot differentiate the insulin response of the participants with IGT from the participants with T2D.

In addition to walking and cycling, another efficacious modality of post-meal physical activity is stair climbing and descending. Bartholomae et al. had adults with pre-diabetes climb and descend stairs for 1, 3, and 10 min at 54%–59% of their respective peak oxygen consumption (VO_2peak_), or about 58%–74% of their respective peak heart rate (HR_peak_), within 0.5 h of ingesting 75 g dextrose. The 1, 3, and 10 min stair phases dampened peak glucose by a mean of 12, 15, and 35 mg/dL, respectively, compared to an inactive control phase (*p* < 0.001). Moreover, the 3 and 10 min stair phases attenuated 1-h glucose AUC by a mean of 502 ± 1141 and 866 ± 1123 mg/dL/min, respectively, compared to an inactive control phase (*p* = 0.023 and *p* < 0.000) [65]. Takaishi et al. had adults with pre-diabetes climb and descend stairs for 6 min at ~60% of their respective HRR, 1.5 h after starting lunch. The 6 min stair phase blunted glucose at 1.75 and 2 h compared to an inactive control phase [67]. In another clinical trial (discussed in the preceding paragraph about cycling), Takaishi and Hayashi had adults with IGT climb and descend stairs for ~8 min at ~60%–65% of their respective HRR, 1.5 h after starting breakfast [66]. With stair activity, participants’ blood glucose was lower at 1.75 and 2 h compared to when they completed an inactive control phase (1.75 h: −27.0%; 2 h: −22.3%) [66]. Stair climbing and descending also facilitated a 356% greater clearance of glucose than an inactive control phase between 1.5 and 1.75 h after the dietary challenge [66]. While Bartholomae et al. did not report insulin values [65], Takaishi et al. reported that postprandial insulin was not significantly different between the stair phase and inactive control phase (*p* > 0.05) [67]. Takaishi and Hayaishi reported insulin data for a sample comprised of participants with either IGT or T2D, but there was not a significant difference between the cycling, stairs, and the control phases (*p* > 0.05) [66].

### 3.5. Effects of Physical Activity on Postprandial Markers of Oxidative Damage and Inflammation

The only clinical trial in this review that evaluated the effect of post-meal physical activity on markers of oxidative damage and inflammation was the walking clinical trial that Diekmann et al. conducted [60]. In older, obese adults with IFG who walked for 30 min after a dietary challenge, 4.5-h IL-6 AUC was greater compared to when they completed an inactive control phase (403.5% greater after first walking vs. first control phase; 164.3% greater after second walking phase vs. second control phase, *p* = 0.035). The 4.5-h Vitamin C AUC was also greater after walking compared to control (23.0% after first walking phase vs. first control phase; 353.9% after second walking phase vs. second control phase, *p* = 0.002). On the other hand, concentrations of OxLDL, sICAM-1, sVCAM-1, sE-selectin, retinol, α-tocopherol, and β-carotene were not significantly different between walking and control phases (*p* > 0.05) [60].

## 4. Discussion

Our primary aim in writing this comprehensive review was to summarize the findings of controlled clinical trials on the effects of acute dietary polyphenol supplementation and post-meal physical activity on postprandial metabolism in adults with features of MetS. Our secondary aim was to recommend changes to current health guidelines for U.S. adults. This population-level guidance is for preventing dysmetabolism and chronic diseases but does not specifically identify valuable sources of dietary polyphenols to include in the diet or promote physical activity shortly after a meal. Augmenting the guidelines to delineate precise recommendations may help the 22% of U.S. adults who presently meet the MetS criteria [2] and support efficacious lifestyle therapies. Controlling PPG is particularly important due to the strong link between postprandial dysglcyemia and CVD [16,22,23]. Available data show that regular dietary polyphenol consumption attenuates PPG, postprandial TG, and markers of oxidative damage in adults with features of MetS [28,31], and that physical activity after a dietary challenge attenuates PPG in T2D [51]. Our review is novel and contributes to the field because it offers, for the first time, a summary of acute interventions with diet and physical activity in adults with features of MetS. In this way, our review offers new insight into treatments for postprandial dysmetabolism in MetS, the global health concern of our time.

### 4.1. Dietary Polyphenols from Oils, Fruits, Teas, and Legumes

The ability of extra virgin olive oil to reduce postprandial ApoB48, gut-derived LPS, and OxLDL is of clinical value. ApoB48 is the key apolipoprotein of the chylomicrons formed in the small intestine that transfer lipids, particularly TGs, to the bloodstream [70]. Peaked levels and prolonged postprandial apoB48 excursions are common in T2D, caused by increased intestinal synthesis and impaired lipolytic clearance. Chylomicrons thus remain in the circulation, where they are lipolyzed into TG-rich lipoproteins. Some of these molecules translocate through to the endothelium into the vascular wall and are phagocytized by arterial macrophages, forming foam cells characteristic of CVD [15,71]. Reducing postprandial LPS is also important because LPS triggers endotoxemia and oxidative stress, particularly in patients with T2D who exhibit an exaggerated response to a high-fat meal [72]. The reduction in both ApoB48 and LPS suggests that extra virgin olive oil may impair chylomicron formation and thus LPS translocation from the gut [52]. The ability of extra virgin olive oil to attenuate oxidative stress is also supported by the lower OxLDL concentration. OxLDL is implicated in atherogenesis [73] and correlates directly with TG, homeostatic model assessment of insulin resistance, and glycated hemoglobin (HbA1c) in patients with T2D and CVD [74].

The relatively larger number of clinical trials investigating fruit-derived dietary polyphenols allows for a deeper discussion and comparisons among the trials. Supplementation of 60 g whole grape powder decreased ET-1, a vasoconstrictor, but this was the first clinical trial to evaluate ET-1 after supplementing grape polyphenols to a high-fat, high-carbohydrate meal [53]. Interestingly, red wine polyphenols added to bovine aortic endothelial cells in vitro inhibited the transcription of the *ET-1* gene [75]. Certainly, more clinical trials are needed to verify whether this mechanism is responsible for the reduced postprandial ET-1 in humans after consuming grape polyphenols. In addition to their effects on ET-1, polyphenols from 300 mg grape seed extract lowered 6-h glucose and OxLDL at 5 h [58]. The reduction in glucose with no difference in 6-h insulin suggests that grapes may improve PPG by improving the efficiency of insulin signaling pathways [58].

It is surprising that 300 mg grape seed extract, but not the 60 g of grape powder, reduced PPG. The extract contained only 94.3 gallic acid equivalents of total polyphenols, while the powder contained 297 gallic acid equivalents [53,58]. The null finding with the powder despite its greater dose of polyphenols may have been due to participants’ characteristics. Only 12 of the 20 participants who received the powder had MetS [53], whereas all the participants who received the extract had MetS [58]. Another consideration is that, compared to the extract, the powder accompanied a dietary challenge denser in both energy (~1035 vs. ~670 kcal) and fat (~54 vs. ~30 g fat) [53,58]. Any protective effect on PPG or PPL by the polyphenols may have been nullified by the considerable systemic challenge.

Resveratrol and curcumin are flavonoids, a class of bioactive dietary molecules shown to benefit metabolic processes [76,77,78]. However, the supplementation of two capsules containing 200 mg resveratrol and 100 mg curcumin did not affect 6-h glucose or insulin in a sample of older adults [54]. A majority of these participants were overweight according to BMI (≥25.0 and <30.0 kg/m^2^) and obese according to waist circumference but were normoglycemic. Only seven participants had MetS [54]. Another important note is that this clinical trial provided a milkshake with ~1110 kcal and 75 g fat [54], a dietary challenge with a similar energy and fat profile as the one used in the clinical trial with grape powder [53]. For reasons already described, a dietary challenge of this caloric and fat load may simply instigate a PPG and PPL response that is unaffected by dietary polyphenols. On the other hand, resveratrol and curcumin did lower 6-h sVCAM-1 compared to the control. Elevated sVCAM-1 is a strong predictor of fatal cardiac events in patients with coronary artery disease (CAD) [79]. Thus, the blunted sVCAM-1 expression after resveratrol and curcumin suggests that supplementation attenuates postprandial inflammation.

Among the other dietary fruits covered in this review are strawberries, a type of commonly consumed berry fruit that appears to benefit postprandial metabolism. Strawberries contain several dietary polyphenols, including flavonols, phenolic acids, ellagitannins, and anthocyanins, in addition to essential micronutrients [80]. Many of these bioactive components are retained when strawberries are preserved via freeze-drying, where the fruit is dried at very low temperatures [81]. The lower 6-h insulin but similar glucose concentrations following consumption of freeze-dried strawberry powder [56,68] suggests that freeze-dried strawberries improved insulin sensitivity, specifically the efficiency of insulin to signal the uptake of a given glycemic load. This assertion seems to have been corroborated by in vitro experiments where skeletal muscle cells under metabolic stress were treated with an extract of the same freeze-dried strawberry powder provided to humans in Park et al.’s in vivo clinical trial [56]. Apparently, in the skeletal muscle cells, phosphorylation of the inhibitory serine residue of the insulin receptor substrate-1 was reduced, and phosphorylation of the stimulatory tyrosine residue was increased. The authors further stated that the activity of the insulin receptor and protein kinase B/phosphatidylinositol 3-kinase pathways were improved (cited and discussed by Park et al. [56], but limited to abstracts for these findings). Nevertheless, the proposed mechanisms align with recent reviews of mechanisms by which berry polyphenols improve dysglycemia [30,82]. In contrast to the agreement on postprandial insulin between the clinical trials, postprandial TGs were unaffected by supplementation in one clinical trial [56], but were reduced in another [69]. In the latter, the participants were overweight and had hyperlipidemia [69] as opposed to obesity and insulin resistance [56]. It may be that the benefits of strawberry polyphenols are limited to people with abnormally elevated blood lipids. The relationship between the acute ingestion of strawberries and postprandial TG requires further attention, given the link between TG and CVD [15,24,25].

Strawberry polyphenols may improve postprandial oxidative damage and inflammation. Although Park et al. did not observe a significant effect on IL-6 or ORAC [56], Edirisinghe et al. reported lower IL-6 and hs-CRP after consuming freeze-dried strawberries compared to the control phase [68]. The lower postprandial hs-CRP is a notable finding because a higher hs-CRP concentration is associated with an elevated risk of T2D, CAD, ischemic stroke, heart failure, and mortality [83]. Strawberry polyphenols also suppressed OxLDL (normalized to fasting) in both adults with obesity and IFG [56] and overweight adults with hyperlipidemia [69]. Dietary polyphenols from strawberries may bind to LDL particles and inhibit their modification by reactive oxygen species [69], thereby attenuating postprandial oxidative damage and protecting the vasculature.

We found a single clinical trial that reported the postprandial effects of acutely ingesting black tea polymerized polyphenols. Both the 110 and 220 mg doses lowered postprandial glucose without changing postprandial insulin [64]. In vitro studies with rabbit and human intestinal cells have shown that tea catechins, including those from black tea, inhibit glucose uptake from the gut [84]. In vivo clinical trials also indicate that black tea benefits PPG. An extract of black, green, and mulberry tea induced carbohydrate malabsorption of 25% compared to a control phase in healthy humans, possibly by inhibiting the gut enzymes α-amylase and α-glucosidase and the gut sodium–glucose transporters [85]. In another clinical trial with healthy humans, 1 g of instant black tea with a 75 g glucose OGTT lowered PPG at 2 h compared to negative (just water) and positive (water with caffeine) control phases [86]. The extract also increased insulin at 1.5 h compared to both control phases [86]. Collectively, these findings suggest that black tea inhibits PPG primarily by interfering with carbohydrate digestion and absorption, but also by potentially improving insulin output by pancreatic β-cells.

Finally, we report the role of black beans in lowering insulin and improving antioxidant activity [55,57]. These findings are congruous with published literature. Black beans may reduce insulin and raise antioxidant capacity due to their anthocyanins, a class of dietary polyphenols [87]. Habitual intake of anthocyanins is associated with better insulin sensitivity and inflammation in women [88] and better glycemic control, insulin sensitivity, and antioxidant capacity in adults with T2D [89]. In the context of acute supplementation, beans also show benefits for metabolism. In adults with T2D, pinto and black beans attenuated postprandial 3-h glucose AUC compared to a control meal of white long-grain rice [90], and in healthy adults, an extract of the common bean (*Phaseolus vulgaris*) improved postprandial glucose, insulin, and C-peptide [91]. These data justify additional clinical trials with adults with MetS to determine the postprandial effects of acute black bean supplementation.

### 4.2. Walking, Cycling, and Stair Climbing and Descending

Physical activity heightens the activity and energy expenditure of skeletal muscle activity above its resting level, necessitating the delivery, uptake, and oxidation of energy substrates such as lipids (i.e., TG and fatty acids) [92,93] and glucose [40,41,43], from the blood. Regarding glucose in particular, the contraction of skeletal muscle in animal models enhances glucose uptake via glucose transporters, especially GLUT4, in both an insulin-dependent [94,95,96] and insulin-independent, contraction-stimulated [97,98,99] fashion. Human skeletal muscle operates similarly, expediting the clearance of glucose from the blood during physical activity in both healthy people and people with obesity and T2D [38,39,40,41,43]. This mechanism is likely responsible, at least in part, for the marked reduction in PPG that we observed in the clinical trials summarized in the present review and discussed next. Although blood lipids and glucose both play important roles in health and postprandial metabolism, hereafter we place a special focus on PPG. This is because (1) in the treatment of MetS and T2D, the target is restoring glycemic control to reduce cardiovascular risk, and (2) few clinical trials with post-meal physical activity have reported PPL or markers of oxidative damage and inflammation. Where such values have been reported, we have discussed them and provided context.

In the clinical trial reported by Lunde et al., the reduction in PPG indicates that ambulatory adults with features of MetS can protect their health with post-meal walks. Participants walked in groups while conversing. As such, each participant self-selected and varied her speed throughout the 20 or 40 min to maintain what the person perceived as a comfortable stroll [62]. Excitingly, adults may extend this benefit to improve their overall daily glycemic load by walking after multiple meals [61]. Importantly, researchers observed the benefits of walking at a speed of just ~4.8 km/h (just under 3 mph and approximately 3.0 metabolic equivalents) [61], which for many people requires just a low-to-moderate-intensity effort [100]. This level of effort is well-suited for “physical activity snacks”, which ideally could be planned or spontaneous, completed without special attire, would not be exhausting, and would not cause excessive sweating (depending on the climate). Further, a daily 45 min walk throughout the week would help U.S. adults meet the 150–300 min of recommended physical activity per week [100,101].

The trial by Diekmann et al., on the other hand, reported null findings on postprandial glucose and lipids following post-meal walking [60]. This may have been caused by the high caloric density of the dietary challenge. Lunde et al. provided participants with 50 g of available carbohydrate [62], and DiPietro et al. provided 1/3 of participants’ respective daily caloric intake (32 kcal/kg of body mass) [61]. In contrast, Diekmann et al. provided participants ~1115 kcal [60], which may have generated such exaggerated PPG and PPL that any activity-induced glucose or lipid clearance was insufficient to generate a statistically significant difference from the control phase. Postprandial IL-6 was higher after walking in this trial, possibly due to a systemic pro-inflammatory response to the meal or an anti-inflammatory response to walking by skeletal muscle [60]. High-carbohydrate, high-fat meals cause the release of IL-6 as an inflammatory marker, but IL-6 is also released by contracting skeletal muscle as a putative anti-inflammatory myokine [60,102]. The higher postprandial vitamin C may be explained by the greater glucose concentration at 1.5 h after walking [60]. Glucose and vitamin C share similar molecular transport pathways, and so a greater PPG concentration that reached significance at 1.5 h after walking may have caused the release of intracellular vitamin C [103,104]. Although IL-6 and vitamin C were increased with walking, it is unclear whether walking heightens or attenuates the overall postprandial oxidative inflammatory states. Given that OxLDL, sICAM-1, sVCAM-1, sE-selectin, retinol, α-tocopherol, and β-carotene did not differ [60], it seems that most likely the oxidative and inflammatory states were largely unaffected. The absence of a walking-induced increase in OxLDL contrasts with evidence that a bout of aerobic exercise acutely raises OxLDL in adults with atherogenic risk (i.e., hypertensive and hyperlipidemic) [105]. It is possible that 30 min of light-to-moderate walking does not generate enough oxidative stress to promote significantly higher oxidation of LDL-C.

While HR, VO_2max_, and VO_2peak_ were not reported in the walking trials, cycling presumably required a greater absolute and relative intensity of physical effort. Importantly, intensity is a key determinant of glucose uptake by skeletal muscle [43]. It is likely the greater intensity of cycling that caused the substantial reduction in PPG in a short period of time. For example, PPG declined by 20% after eight min of cycling compared to an inactive control phase [66]. While not statistically significant in the clinical trial [66], a decrease of this size in practice could normalize a borderline high PPG. Furthermore, a 20% reduction in PPG is a clinically meaningful finding, given the progressive positive relationship between PPG and the risk of developing CVD [17,18]. It is unclear why TG was unchanged after post-meal cycling, and there are scant studies that report the effects of post-meal cycling on PPL. One explanation may be that cycling occurred too late after the dietary challenge (1 h after) [63]. Another consideration is that the dietary challenge was relatively small, containing only 4.8 kcal/kg of body mass (~326 kcal for a 68 kg participant) and 9% fat. In the example of the 68 kg participant, the person would have only ingested ~3 g of fat. The Western diet is characterized by meals containing a far greater percentage of fat [106]. Excess dietary fats contribute to PPL and thus may require greater intensity or duration of post-meal exercise to reveal a difference.

As with cycling, climbing and descending stairs requires a greater effort than walking, largely due to the vertical component. Participants who climbed and descending stairs worked at relative intensities (e.g., %VO_2peak_, %HR_peak_, %HRR) comparable to participants in the cycling clinical trials (Table 2). Importantly, 1–10 min of the activity improved PPG [65,66,67]. Only Takaishi et al. reported postprandial insulin, which was not different from the inactive control phase [67]. This finding points to stair climbing and descending provoking insulin-independent glucose uptake, which has favorable clinical implications. These findings highlight the importance of taking the stairs rather than the elevator in one’s daily life.

In summary, post-meal walking, cycling, and stair climbing and descending, initiated 0.5–1.5 h after a dietary challenge, efficaciously blunt PPG in adults with features of MetS. Furthermore, completing physical activity that is more intense, or accumulating activity throughout the day, may further reduce PPG. To our knowledge, our review is the first to summarize this phenomenon in this population based on clinical data. Our conclusions align with a recent meta-analysis [107] and systematic review [51] highlighting the efficacy of post-meal physical activity in controlling PPG in adults with T2D. The benefits of post-meal physical activity to improve PPG are consistent; however, the effects on PPL and postprandial oxidative damage and inflammation are not conclusive and deserve more attention.

### 4.3. Recommendations

The U.S. Department of Agriculture (USDA) recommends that U.S. adults moderate their caloric intake and consume two 1 cup equivalents of fruit per day for a 2000 kilocalorie diet, preferably as whole fruits [108]. At present, nearly every age demographic between both sexes in the United States fails to meet this recommendation [108]. Among its recommendations for individual fruits, the USDA lists ½ cup of strawberries as a ½ cup equivalent of fruit [108]. Supplementing the diet with whole strawberries is a feasible way by which people can meet the guidelines and obtain the health benefits of fruits. Strawberries are low-calorie (~50 kilocalories per one cup) [109], dense in micronutrients and polyphenols [80], and have been shown to improve postprandial insulin economy and some markers of oxidative damage and inflammation in clinical trials included in this review. Most adults eat frequently throughout the day. Supplementing two to four of those meals with ½ to 1 cup strawberries may provide postprandial health benefits without dramatically increasing total caloric intake.

The U.S. Office for Disease Prevention and Health Promotion recommends completing ≥150 min (2.5 h) of moderate-intensity aerobic activity per week and muscle-strengthening activities on at least two days per week [101]. Achieving these guidelines helps adults stave off risk factors and conditions associated with cancer, stroke, T2D, and CVD: being overweight, obesity, hypertension, and high blood cholesterol and TG [101]. In addition to reducing the risk of disease, physical activity confers benefits to cognition and physical fitness, including improved aerobic capacity, muscular strength, muscular endurance, and balance [101,110]. While informative, the guidelines on physical activity do not provide a directive on when adults should be physically active to optimally improve their health. This comprehensive review suggests that post-meal physical activity may help adults with cardiometabolic risk factors attenuate their postprandial dysmetabolism. Future guidelines must reflect this observation. Post-meal physical activity in the form of walking, cycling, or stair climbing and descending may enable adults with MetS to directly and immediately reduce their PPG excursions, thereby improving an otherwise deleterious postprandial state. In the self-management of MetS, pre-diabetes, and T2D, exercising after a dietary challenge and observing the tangible outcome of a marked reduction in PPG may confer a greater sense of self-efficacy and motivation toward better health.

### 4.4. Strengths and Weaknesses

The present review is strengthened by having clearly defined inclusion and exclusion criteria that were used to complete the literature search (Figure 1). An additional strength is the decision to include only controlled clinical trials for analysis. All clinical trials with dietary polyphenols had a control phase where participants were treated without dietary polyphenols (matched dietary challenge or a placebo). Similarly, all clinical trials with physical activity had an inactive control phase. Another strength is that this review included clinical trials that examined four major sources of dietary polyphenols (oils, fruits, teas, and legumes) in doses achievable in the diet, and three different modalities of physical activity (walking, cycling, and stair climbing and descending) in achievable intensities and durations.

The present review was limited by its inclusion of only clinical trials published in Google Scholar and PubMed. However, these databases are robust repositories for peer-reviewed journal articles that present data from clinical trials. Though the omission of relevant articles is possible, great care was taken to avoid doing so. This review also only includes articles published in English and does not include unpublished clinical trials (e.g., listed on https://www.clinicaltrials.gov, but data have not been disseminated). This review was also limited by the overall characteristics of clinical trials as follows: (1) the small number of controlled clinical trials on tea-, legume-, and oil-derived polyphenols that fit our inclusion criteria, and (2) a disproportionately heavy focus on PPG compared to other postprandial biomarkers, especially those related to postprandial oxidative stress and inflammation that were not reported by the majority of clinical trials in this review. These limitations thus reveal a great need for future research: new clinical trials should measure postprandial insulin, TG, and markers of oxidative damage and inflammation in response to a broader selection of functional foods with or without physical activity in adults with MetS.

## 5. Conclusions

**Figure 2:**
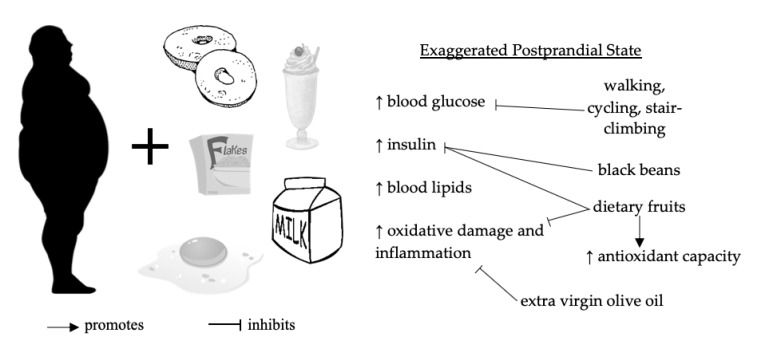
Effects of acute dietary polyphenol supplementation and post-meal physical activity on postprandial metabolism in adults with features of the metabolic syndrome (MetS).

Dietary challenges trigger a dynamic postprandial state wherein organ systems must cooperate to digest and absorb energy substrates from ingested food for storage or use. This state is exaggerated and prolonged in adults with features of MetS or pre-diabetes. Clinical trials summarized in our review demonstrated that acute lifestyle interventions based on diet and physical activity improve postprandial metabolism in this high-risk group (Figure 2). This review expands upon current guidelines by specifying types of dietary fruits (e.g., grapes and strawberries, blackcurrant) and physical activity modalities (walking, cycling, stair climbing and descending), as well as the timing of physical activity (post-meal), that may acutely improve postprandial dysmetabolism in adults with features of MetS. Finally, these conclusions are based on the limited availability of clinical data on postprandial interventions in MetS, which deserve urgent attention in future clinical trials.

## Figures and Tables

**Table 1:** Clinical trials on the effects of acute oil-, fruit-, tea-, and legume-derived dietary polyphenol supplementation on postprandial glucose, insulin, lipids, and markers of oxidative damage and inflammation.

Authors, Year (Country)	Trial Design	Participants ^1^	Intervention and Dietary Challenge	Glucose and Insulin	Lipids	Markers of Oxidative Damage and Inflammation
Carnevale et al., 2019 (Italy) [52]	Randomized crossover	Obese adults with IFG (*n* = 30, age = 58 ± 11)	10 g EVOO Test meal (~725–750 kcal, 28%–30% fat, 53%–54% CHO, 16%–19% PRO)	NR	↓ ApoB48 at 2 h	↓ LPS at 1 h and 2 h ↓ OxLDL at 1 h and 2 h ↓ sNox2-dp at 1 h and 2 h
Bardagjy et al., 2018 (USA) [53]	Randomized crossover	Obese adults (*n* = 20, 12/20 with MetS, age = 49 ± 15 years)	60 g GP Test meal (~1035 kcal, 47% fat, 41% CHO, 12% PRO)	NS 5-h glucose iAUC, 5-h insulin iAUC	NS 5-h TG iAUC	NS IL-6, MCP-1, OxLDL, PAI-1, RBP4, sICAM-1, sVCAM-1, TNF ↓ ET-1 at 5 h
Vors et al., 2018 (Canada) [54]	Randomized crossover	Older adults (*n* = 22, 7/22 with MetS, age = 53–70 years)	Res + Cur (200 mg Res + 100 mg Cur) Homogenized milkshake (~1110 kcal, 75 g fat, 60% fat, 25% CHO, 15% PRO)	NS 6-h glucose iAUC, 6-h insulin iAUC	NS 6-h TG iAUC	NS 6-h iAUC for IL-6, IL-8, MCP-1, CRP, sICAM-1, sE-selectin ↓ 6-h sVCAM-1 iAUC
Butacnum et al., 2017 (Thailand) [64]	Randomized crossover	Adults with pre-diabetes (*n* = 11, age = 45 ± 10 years)	500 mL black tea with low and high dose of BTPP (110 and 220 g, respectively) 50 g sucrose in 200 mL water	↓ 1-h and 1.5-h glucose iAUC (110 and 220 mg BTPP) NS insulin	NR	NR
Reverri et al., 2017 and Reverri et al., 2015 (USA) [55,57]	Randomized crossover	Adults with MetS (*n* = 12, age = 49 ± 14 years)	BB, AF, or NF Test meal with BB, AF, or NF (~930 kcal, 25 g fat)	NS glucose ↓ 5-h insulin (BB vs. AF and NF)	NS TG	NS IL-6, OxLDL, sICAM-1, sVCAM-1
Park et al., 2016 (USA) [56]	Randomized crossover	Obese adults with IFG (*n* = 21, age = 40 ± 14 years)	0, 10, 20, or 40 g FDS Bagel, cream cheese, margarine, hard-boiled egg, cantaloupe, and whole milk with strawberry beverage (~975 kcal, 25 g fat)	NS glucose ↓ 6-h insulin (40 g FDS vs. 0 g and 10 g FDS) ↓ insulin absolute peak and incremental increase from baseline (40 g FDS) ↓ I:G ratio (40 g vs. 0 g and 10 g FDS)	NS TG	NS IL-6, ORAC ↓ 6-h OxLDL (normalized to fasting; 20 g vs. 40 g, 10 g, and 0 g FDS)
Edirisinghe et al., 2012 (USA) [58]	Randomized crossover	Adults with MetS (*n* = 12, age = 45 ± 15 years)	300 mg GSE Bagel, cream cheese, margarine, egg, cantaloupe, and whole milk (~670 kcal, 30 g fat)	NS 6-h insulin AUC ↓ 6-h glucose AUC	NS 6-h TG AUC, 6-h cholesterol AUC	NS 6-h IL-6, TNF-α, lipophilic ORAC iAUC ↓ OxLDL at 5 h vs. baseline ↑ 6-h hydrophilic ORAC iAUC
Huebbe et al., 2012 (Germany) [59]	Crossover	Adult males with atherosclerosis-prone phenotype (*n* = 11, age = 37 ± 6 years)	250 g BC beverage 200 g cream (30% fat) with 75 g sucrose	NS glucose, insulin	NS TG, TC, LDL-C, HDL-C	NS IL-6, IL-1β (ex vivo), OxLDL, α-tocopherol, PON ↑ IL-6 at 4 h compared to baseline ↓ IL-1β and TNF-α (ex vivo) at 4 h vs. baseline (PBO) ↑ ORAC at 1.5 h and 2 h ↑ 2-h and 4-h ORAC AUC ↑ ascorbic acid at 2 h, 2.5 h, 3 h, 3.5 h, and 4 h ↑ 4-h ascorbic acid AUC
Edirisinghe et al., 2011, and Burton-Freeman et al., 2010 (USA) [68,69]	Randomized crossover	Overweight, hyperlipidemic adults (*n* = 24, age = 51 ± 15 years)	10 g FDS Bagel, cream cheese, margarine, hard-boiled egg, cantaloupe, whole milk, and milk-based strawberry beverage (~960 kcal, 31 g fat)	NS glucose ↓ 6-h insulin and at 1 h and 3 h	↓ 6-h TG and at 4 h and 5 h ↑ 6-h LDL-C in men	NS PAI-1, TNF-α, IL-1β ↓ 6-h IL-6 and at 6 h ↓ 6-h hs-CRP ↓ 6-h OxLDL (normalized to fasting) in men

^1^ Mean ± standard deviation (SD); impaired fasting glucose; EVOO: extra virgin olive oil; kcal: kilocalories; CHO: carbohydrate; PRO: protein; NR: not reported; ApoB48: apolipoprotein B48; PBO: placebo treatment; LPS: lipopolysaccharides; h: hour; OxLDL: oxidized low-density lipoprotein cholesterol; sNox2-dp: soluble Nox2-derived peptide; United States of America; MetS: metabolic syndrome; GP: whole grape powder; NS: non-significant difference between treatments; h: hour; iAUC: incremental area under the curve; TG: triglycerides; ET-1: endothelin-1; PBO: placebo treatment; IL-6: interleukin-6; MCP-1: monocyte chemoattractant protein-1; OxLDL: oxidized low-density lipoprotein cholesterol; PAI-1: plasminogen activator inhibitor-1; RBP4: retinol-binding protein 4; sICAM-1: soluble intercellular adhesion molecule-1; sVCAM-1: soluble vascular cell adhesion molecule-1; TNF: tumor necrosis factor; Res: resveratrol; Cur: curcumin; g: grams; IL-8: interleukin-8; CRP: C-reactive protein; sE-selectin: soluble endothelial selectin; mL: milliliters; BTPP: black tea polymerized polyphenols; BB: black beans; AF; added fiber; NF: no fiber; IFG: impaired fasting glucose; FDS: freeze-dried strawberries; I:G ratio: insulin-to-glucose ratio; ORAC: oxygen radical absorbance capacity; FM: fiber-matched; AM: antioxidant-matched; GSE: grape seed extract; AUC: area under the curve; BC: blackcurrant; TC: total cholesterol; HDL-C: high-density lipoprotein cholesterol; IL-1β: interleukin-1β; PON: paraoxonase; hs-CRP: high-sensitivity C-reactive protein.

**Table 2:** Clinical trials on the effects of acute post-meal physical activity on postprandial glucose, insulin, lipids, and markers of oxidative damage and inflammation.

Authors, Year (Country)	Trial Design	Participants ^1^	Dietary Challenge and Intervention	Glucose and Insulin	Lipids	Markers of Oxidative Damage and Inflammation
Diekmann et al., 2019 (Germany) [60]	Randomized crossover	Older obese adults with dyslipidemia, IFG, or inflammation (*n* = 26, age = 70 ± 5 years)	Test meal (~1115 kcal, 40–59 g fat) 30 min walking (4.6 ± 0.1 km/h, ~12 RPE) immediately after test meal	NS 4.5-h glucose AUC, 4.5-h insulin AUC ↑ glucose at 1.5 h ↓ insulin at 3 h	NS TG, NEFA AUC	NS OxLDL, sICAM-1, sVCAM-1, sE-selectin, retinol, α-tocopherol, β-carotene ↑ 4.5-h IL-6 AUC ↑ 4.5-h Vitamin C AUC
Bartholomae et al., 2018 (USA) [65]	Randomized crossover	Adults with pre-diabetes (*n* = 30, 26 ± 6 years)	Dietary challenge: 75 g dextrose OGTT 1, 3, or 10 min stair climbing and descending (54%–59% VO_2peak_/58%–74% HR_peak_) at 27, 25, and 18 min, respectively, after OGTT	↓ peak glucose at 0.5 h (1, 3, and 10 min)) ↓ 1-h glucose AUC (3- and 10-min)	NR	NR
Takaishi & Hayashi, 2017 (Japan) [66]	Randomized crossover	Adults with IGT (*n* = 7, 51 ± 3 years)	Test meal (~660 kcal, 18 g fat) ~8 min stair climbing and descending vs. cycle ergometry (both modalities at 60%–65% HRR), 90 min after starting meal	↓ glucose at 1.75 h and 2 h (stair climbing and descendingbut not cycling) ↑ glucose clearance between 1.5 h and 1.75 h (stair climbing and descending but not cycling) ↑ net glucose clearance between 1.5 h and 1.75 h (stair climbing and descending vs. cycling) ^2^	NR	NR
DiPietro et al., 2013 (USA) [61]	Randomized crossover	Older adults with IFG (*n* = 10, age = 69 ± 6 years)	Three test meals [(~32 kcal/kg body mass) across 3 meals, 31% fat) 15 min walking (4.8 ± 0.6 km/h, 3 METs), 30 min after breakfast, lunch, and dinner (3 total bouts during the day)	↓ 24-h glucose AUC ↓ 3-h post-dinner glucose AUC	NR	NR
Takaishi et al., 2012 (Japan) [67]	Randomized crossover	Adult males with pre-diabetes (*n* = 8, age = 48 ± 7 years)	Test meal (~660 kcal, 18 g fat) 6 min stair climbing and descending (~60% HRR, 13 RPE), 90 min after starting meal	NS insulin ↓ glucose at 1.75 h and 2 h	NR	NR
Lunde et al., 2012 (Norway) [62]	Crossover	Obese adult females (*n* = 11, 5/11 with IGT) age = 44 ± 9 years)	Corn flakes with milk (50 g available CHO) 20 min or 40 min walking (self-selected pace) immediately after a meal	↓ peak glucose (40 min walking) ↓ 2-h glucose iAUC (20 and 40 min walking)	NR	NR
Derave et al., 2007 (Belgium) [63]	Randomized crossover	Sedentary adult males with MetS (*n* = 7, age = 45 ± 11 years)	Test meal (~4.8 kcal/kg body mass, 9% fat, 82% CHO, 9% PRO) 45 min cycle ergometer (60% VO_2max_), 60 min after starting breakfast	↓ glucose at 0.75 h and 1 h after start of physical activity	NS TG iAUC	NR

^1^ Mean ± standard deviation (SD); ^2^ Change in glucose between 1.5 and 1.75 h after treatment minus the change in glucose between 1.5 and 1.75 h during control; *n*: sample size; yrs: years; min: minutes; km/h: kilometers per hour; RPE: Borg’s Rating of Perceived Exertion; kcal: kilocalories; g: grams; NS: non-significant findings between treatments; h: hours; AUC: area under the curve; CON: control; TG: triglycerides; NEFA: non-esterified fatty acids; IL-6: interleukin-6; OxLDL: oxidized low-density lipoprotein cholesterol (LDL-C); sICAM-1: soluble intercellular adhesion molecule-1; sVCAM-1: soluble vascular cellular adhesion molecule-1; sE-selectin: soluble endothelial selectin; USA: United States of America; VO_2peak_: peak oxygen consumption; HR_peak_: peak heart rate; OGTT: oral glucose tolerance test; NR: not reported; IGT: impaired glucose tolerance; HRR: heart rate reserve; IFG: impaired fasting glucose; MET: metabolic equivalent; kg: kilograms; CHO: carbohydrate; iAUC: incremental area under the curve; MetS: metabolic syndrome; PRO: protein.
